# Anomaly detection in microservice environments using distributed tracing data analysis and NLP

**DOI:** 10.1186/s13677-022-00296-4

**Published:** 2022-08-13

**Authors:** Iman Kohyarnejadfard, Daniel Aloise, Seyed Vahid Azhari, Michel R. Dagenais

**Affiliations:** 1 Department of Computer and Software Engineering, Polytechnique Montreal, Montreal, Canada; 2 Ciena Inc., Ottawa, Canada

**Keywords:** Performance monitoring, Anomaly detection, Tracing, Microservices, Machine learning, NLP, LSTM

## Abstract

In recent years DevOps and agile approaches like microservice architectures and Continuous Integration have become extremely popular given the increasing need for flexible and scalable solutions. However, several factors such as their distribution in the network, the use of different technologies, their short life, etc. make microservices prone to the occurrence of anomalous system behaviours. In addition, due to the high degree of complexity of small services, it is difficult to adequately monitor the security and behavior of microservice environments. In this work, we propose an NLP (natural language processing) based approach to detect performance anomalies in spans during a given trace, besides locating release-over-release regressions. Notably, the whole system needs no prior knowledge, which facilitates the collection of training data. Our proposed approach benefits from distributed tracing data to collect sequences of events that happened during spans. Extensive experiments on real datasets demonstrate that the proposed method achieved an F_score of 0.9759. The results also reveal that in addition to the ability to detect anomalies and release-over-release regressions, our proposed approach speeds up root cause analysis by means of implemented visualization tools in Trace Compass.

## Introduction

Nowadays, computing infrastructures have evolved significantly using complex systems that facilitate many complicated and large-scale tasks in distributed and cloud environments. The *microservice* architecture has emerged as a result of this development. Microservices are small interconnected services that present a complex service, such as a web application [1]. They provide greater scalability, making possible the distribution of an application over multiple physical or virtual systems. In addition, the microservice architecture improves productivity by decomposing applications into smaller services that are easier to manage and faster to develop. Unlike the monolithic architecture, if one microservice fails the others continue to work.

These improvements have increased user expectations in a way that any performance anomaly may lead to user dissatisfaction and loss of revenue. Even when several services are brought down for maintenance, the users usually do not notice it. Although significant efforts have been made to ensure the quality of microservices, the complexity and large scale of these systems make them fragile and prone to performance anomalies and failures [2]. Further, performance monitoring and tracing of microservices become even more challenging as the degree of automation and distribution is increased. For example, each service can be developed using its own language or technology while still communicating with other services.

Unlike monolithic applications in which dedicated teams work on discrete functions such as UI or database, microservices employ cross-functional teams to handle an application’s entire life cycle using a continuous delivery model [1, 3]. Nonetheless, dynamic services make monitoring more difficult. Even if a tracer can record all the execution details, it is still hard to detect the source of the problem inside the trace files.

Different reasons may cause performance anomalies in microservice environments [4]. Any problem in a service, such as a network disconnection or hard disk failure, may cause the microservice system to crash. Misconfigurations or extreme load by a service can also affect the whole system. Changes in one service may influence other dependent services workload, and may result in response time degradation. Moreover, the agile nature of microservice environments yields multiple service updates per day, and several versions of the application may be deployed in a short amount of time. As such, several methods may change in a new update, then affecting the response time behavior of services and leading to many false alarms from monitoring tools [4, 5].

The way we trace such environments and collect data is of particular importance. A microservice-based application consists of tens, hundreds, or thousands of services running across many hosts. Consequently, it is not possible to rely on an individual trace. In this case, distributed tracing is required, thus providing a view of a request’s life as it travels across multiple nodes and services communicating over various protocols [6]. It also enables to follow the spans and events that occur in different nodes. A *span* is the primary building block in distributed tracing and represents an individual unit of work done in a distributed system. Besides, many sub-spans may be generated during the spans lifetime, in which tens of userspace and kernel events occur in a particular order. The proposed diagnostic approach proposed here works by collecting sequences of events during spans using the Linux Trace Toolkit Next Generation (LTTng) [7], sending them to the detection module, and eventually analyzing the outputs of the model in Trace Compass. LTTng provides a system software package for correlated tracing of the Linux kernel, applications, and libraries [7].

In this paper, we propose a general framework to find anomalies as well as release-over-release regressions in microservice environments by taking advantage of NLP and open-source tools (i.e., LTTng and Trace Compass). In general, anomaly detection and localization is the process of finding patterns in data that deviate from normal behavior [8], which is different from noise detection and noise elimination that refer to unwanted noise in the data. Anomalies in data may happen in various forms, such as point and collective anomalies. Methods that work based on detecting point anomalies and also metric-based algorithms cannot always identify the root cause of anomalies. A single data point (event or metric) does not include enough information to determine whether an anomaly actually happened in complex systems such as microservices. Usually, an anomaly is declared when the execution of the program has not been normal during a time interval that includes many events. In real applications, only a limited number of events can be the result of an action. Therefore, just a few of the possible events can appear as the next events for a sequence of observed events [9]. In other words, similar to words in natural language processing, events as elements of a sequence follow specific patterns and grammar rules. We used this idea in our anomaly detection framework and applied a general NLP-based strategy to distinguish normal and abnormal patterns in a sequence of events. In addition to locating anomalies, our proposed framework also allows analysts to zoom in the detected anomalous parts of the trace to discover the root cause of the problems.

The main contributions of our work can be summarized as follows:

- Unlike many other methods that use OpenTracing, our anomaly detection framework employs LTTng to perform distributed tracing. OpenTracing is a vendor-agnostic API to help developers easily instrument tracing into their code [10]. A trace in OpenTracing is a directed acyclic graph of spans, and it provides only relationships across microservices. In contrast, LTTng provides details of the programs execution with higher resolution by presenting kernel and userspace events.
- We developed a handcrafted data extraction module in Trace Compass to construct the spans using the request/response events tag. Moreover, the hierarchical structure of these tags helps us to extract subspans. This module is also responsible for converting each span into a sequence of events.
- Our LSTM-based model, designed for post-analysis of traces, learns the normal patterns of events along with their arguments (e.g., event type, tag, and process name). Further, this model is trained to predict the next event’s arguments in addition to the event’s name. Learning and predicting at this granularity sets our model apart from the others found in the literature.
- Our framework makes it possible to examine the system behavior from both the system and service perspectives, which gives the troubleshooter a deep understanding of what happened at the time of an anomaly. The provided visualizations considerably reduce troubleshooting time by highlighting the anomalous parts of the trace and directing the debugger to the most relevant problem sites of interest. Without such visualizations, manually tracking the performance of systems within low-level tracing data, possibly including thousands of events from different spans, is indeed a very exhausting task.
- In addition to anomaly detection, our framework can be applied to identify release-over-release regressions. Finding potential regressions from one release to another is extremely valuable, and conventional performance tests cannot reveal sufficient regressions. Many subtle changes in spans or sequence of events signify a regression that can be captured using our framework.

The rest of the paper is organized as follows. In Section Previous work, related studies are presented. In “Anomaly detection framework” section, we introduce our automatic integrated anomaly detection framework for microservice environments. “Evaluation” section provides the experimental results followed by the conclusions in “Conclusion” section.

## Previous work

In traditional approaches, application performance management (APM) tools that support various measures are utilized to perform resource behavior analysis on microservices [11]. Tracing is another robust and efficient approach for reverse engineering and debugging of complex systems [12]. Many tracers across all software stack layers, and even at the hardware level, have emerged in the last years. Distributed tracing, unlike the most traditional methods that only monitor individual components of the architecture, is applied to complex distributed systems at the workflow level [13]. Tools like OpenCensus and OpenTracing [10] help to record the execution path of each microservice request. Jaeger [14], a popular tool that supports OpenTracing and developed by Uber, has been widely used to automatically collect and store the service call data [15, 16]. Its counterpart Zipkin [17] aids in gathering timing data needed to troubleshoot latency problems in microservice architectures [18, 19]. However, the high-level information that these tools provide is not always sufficient to characterize the execution status of the system since they do not offer kernel events. Thus, tracing with LTTng is a fundamental part of our anomaly detection framework. This open-source tool is implemented for achieving high throughput and includes multiple modules for Linux kernel and userspace tracing, thereby imposing low overhead to the operating system. Besides, this tool can work with a variety of environments, such as monolithic applications, microservices, and IoT devices [20].

The earliest efforts for anomaly detection had used statistical methods [21] where an anomaly score was calculated using a function of abnormality to show the behavior of the application. In [22], CPU performance and network performance metrics in master-slave and nested-container models are compared to provide a benchmark analysis guidance for system designers. However, a live threshold is required given the system’s current state to determine whether the program behavior is normal or abnormal, which is practically impossible to set in real-time. Furthermore, these tools do not provide any details about the application’s execution flow. Several machine learning-based schemes have also been applied to detect anomalies in microservice systems in addition to statistical and metric-based methods. Hierarchical Hidden Markov Models (HHMM) are adopted in [23] to learn a model based on different monitored metrics such as CPU, Memory, and Network to locate anomalous behaviors. Besides, many clustering algorithms, such as k-means, k-medoids, EM clustering, and outlier detection algorithms, have been employed for anomaly detection in microservice environments [24–26]. The main issue with such methods is that they are usually difficult to interpret. Supervised methods such as SVM, Fuzzy Logic, and Neural Networks, which use labeled data, were proposed in [27–29]. In [28], the authors used a SVM to detect DoS attacks in virtualized clouds under a changing environment. In [30], a fuzzy technique was proposed to extract abnormal patterns based on various statistical metrics in which fuzzy logic rules are applied to classify data. However, in practice, the labeling process is highly complicated, or even impossible. Recently, deep learning techniques which do not need labeled data have yielded promising results. The works of [31, 32] propose anomaly detection methods for large cloud infrastructures using long short-term memory (LSTM) neural networks [33] with data from distributed tracing technologies. In that work, a stacked LSTM network model was presented for anomaly detection in time series where the network was trained on non-anomalous data. The drawback of these methods is that many details, including events arguments such as event type, tag, process name, and return value are ignored.

Furthermore, researchers have made much effort to improve anomaly detection by using different data representations and information resources. Tracing data and log, as the most popular information resources, can be represented in the form of an enumerated collection of events sorted by their timestamps [34]. Different works make different uses of this structure. In DeepLog [35], a deep neural network model is proposed to model an unstructured system log as a natural language sequence. In [36], by performing time-series-based forecasting, anomalies on cyclic resource usage patterns are detected. In the sequel, graph representations of the events are obtained from this data and employed to detect critical nodes and design anti-patterns proactively. The authors of [37] designed and developed a simplified MSA application and applied different graph algorithms, then assessing their benefits in MSA analysis. In another article, Tao Wang et al. [38] organized the trace information collected by the OpenTracing tool to characterize processing requests workflow across multiple microservice instances as a calling tree. The proposed approach converts the given trace into the spans and detects performance anomalies using the model of normal key patterns.

Some points distinguish our work from previous related literature. Fistly, unlike traditional approaches, that use application performance management tools that support various metrics (e.g., CPU and memory utilization) to perform resource behavior analysis on microservices, our work’s main source of information is tracing data. Compared to these approaches, our proposed framework is not dependent on the existence of any threshold. Moreover, the metrics used by these approaches do not help to find the cause of the anomaly after detecting it. Tracing provides considerable details about the application’s execution flow and about what exactly happened at the time an anomaly occurred. Secondly, most previous works that make use of tracing data employ OpenTracing-based tools such as Jaeger or Zipkin to perform distributed tracing. Nonetheless, the high-level information that these tools provide about microservices interaction is not always sufficient to characterize the execution status of the system. Our proposed framework employs the LTTng open-source tool, which imposes low overhead on the operating system and presents low-level kernel and userspace tracing. Thirdly, the main drawback of supervised methods is that they require labeled data. The process of labeling data points in terms of performance status is highly complicated and sometimes even impossible. In addition, to collect labeled data related to an application, a very specialized professional is needed. Conversely, clustering approaches are difficult to interpret. We propose in this work an unsupervised method to learn normal execution patterns, since collecting normal data is straightforward and can be done automatically without any supervision. Fourthly, other deep learning-based and NLP-based approaches ignore events arguments in their modeling. Event arguments such as process name, message, and event type contain beneficial details that increase detection quality [39]. We use these arguments to train our model. Then, in the prediction phase, our model predicts the name of the next event as well as its arguments. Finally, previous works from the literature, such as DeepLog, have not presented any solution to analyze the model’s output. However, the use of Trace Compass in our approach enables us to develop analysis scripts and even use many preexisting scripts and visualizations to examine the model’s output more deeply.

## Anomaly detection framework

In this section, we introduce an NLP-based anomaly detection framework for post-analysis of LTTng traces. It is designed to help developers to efficiently find the root causes of abnormal behaviors in microservice environments. We aim to provide a general framework applicable to microservice-based applications with different settings.

**Fig. 1:**
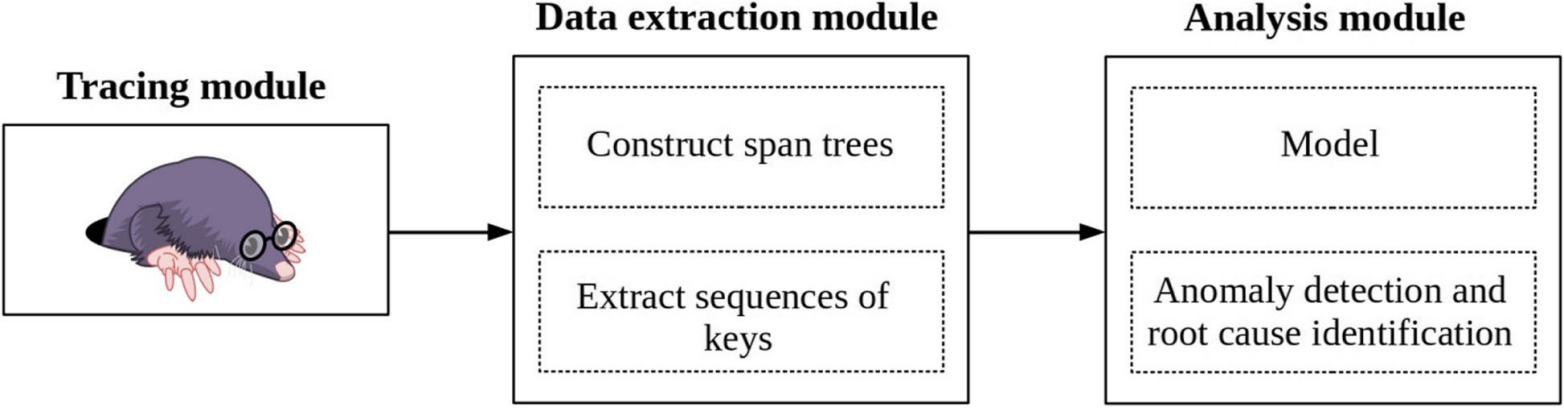
The architecture of our proposed anomaly detection method for microservice environments

Figure 1 presents the architecture of our approach along with its three main modules, i.e., the *tracing module*, the *data extraction module*, and the *analysis module*. We discuss this architecture in detail in the following subsections.

### Tracing module

Tracing is an efficient way of gaining information from a system for further analysis and debugging, thus minimizing the monitoring influence. Distributed tracing is derived from traditional tracing so as to be employed within distributed systems. Distributed tracing technologies provide a view of the communication among microservices [6]. Microservices mostly use Representational State Transfer (REST) as a usual way to communicate with other microservices.

We aim to provide a general anomaly detection framework that can be easily applicable for any microservice-based application in practice and subsequently lead to the discovery of the cause of the identified anomalies. We have described how to analyze an application and prepare its associated dataset, instead of using pre-existing available datasets which do not inherently contain information needed to extract spans and their associated sequence of events.

As our case study, we created our dataset by tracing a distributed software available in Ciena Corporation. Many new releases of this software are provided by the developers of this company every day. Thus, we collected traces from different releases to compose the dataset. We denote the set of all traces collected from different releases as *Γ*={*T*_1_,*T*_2_,...,*T*_*n*_}, where *n* indicates the number of collected traces.

**Fig. 2:**
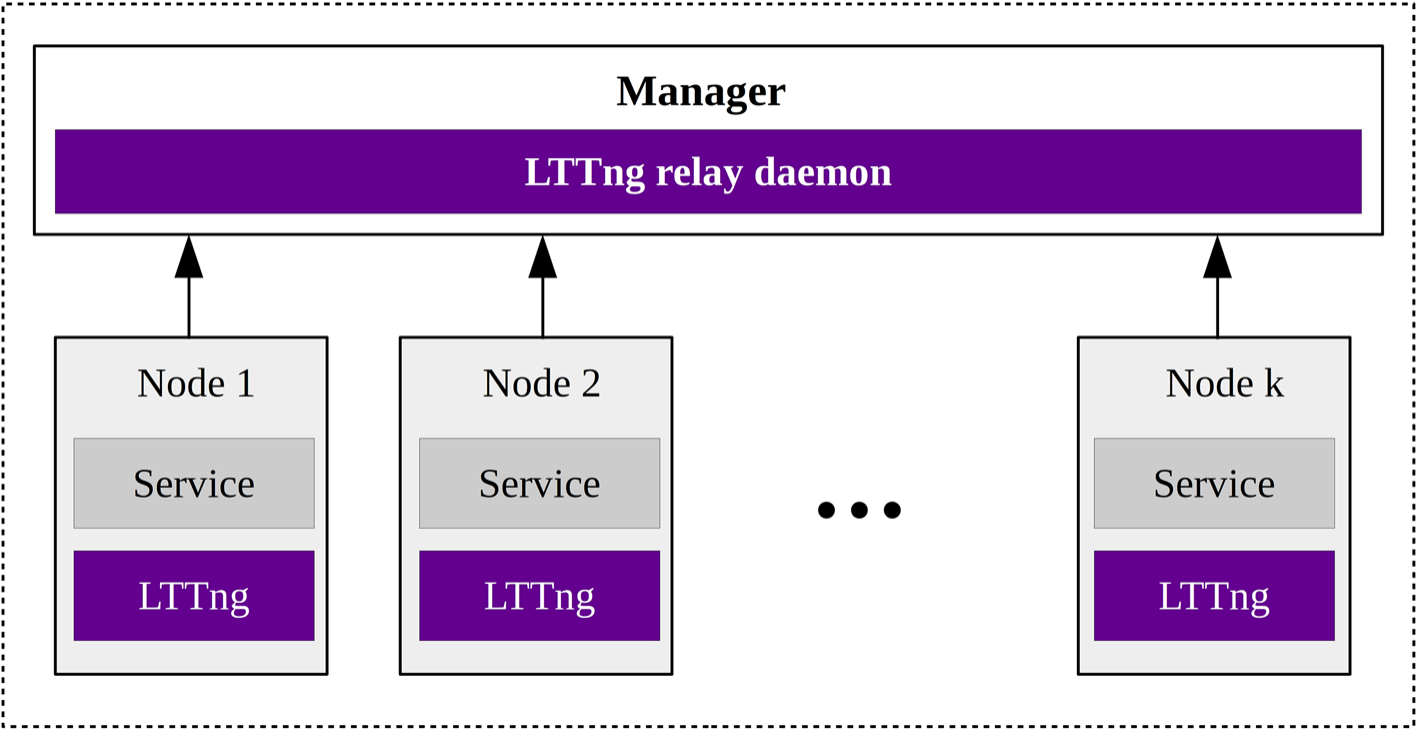
The overview of our distributed tracing module

Figure 2 illustrates the structure of our tracing module that make use of the LTTng open-source tool. As presented in this figure, LTTng is deployed on each node to send the tracing data to the manager. The running LTTng-relayd daemon on the manager collects the tracing data received from the nodes. Later, Trace Compass integrates the traces obtained from different nodes to form a Trace $\phantom {\dot {i}\!}T_{i} = \{e_{1}, e_{2},..., e_{g(T_{i})} \}$, where *g*(*T*_*i*_) is the number of events associated to *T*_*i*_. Actually, *T*_*i*_ is represented as an enumerated collection of events sorted by their timestamps.

During the execution of a microservice application, many tasks or spans, such as opening a web page, are performed. In fact, a trace can be divided into a set of spans, where each span consists of a sequence of events that are invoked in a specific order to perform the desired task. It should be noted that spans can not be directly retrieved using LTTng. In the sequel, we will discuss in detail how to extract spans from tracing data.

### Data extraction module

We implemented the data extraction module within the Trace Compass open-source tool, which offers scripting capability [40] and visualization mechanisms to promote our analysis. LTTng generates a CTF (Common Trace Format) file for every node in the microservice environment. The CTF format is a file format optimized for the production and analyses of big tracing data [7]. After generating the CTF files, Trace Compass is used to read these files and integrates them into trace *T*_*i*_, where *i* indicates the index of this trace in *Γ*. The result of this process is an enumerated collection of events sorted by their timestamps.

An event is composed of well-defined fields that are common to all events, such as name, timestamp, and process ID. However, the delivered sequence of time-ordered events does not provide the spans that reflect separate tasks. In order to extract spans and their subspans, *Γ* is scanned with respect to the tag of request/response events. Other events are then processed, so as that each event is assigned to the span it belongs. In our framework, events are stored by means of their associated *keys* composed by the name of the event and its arguments.

**Fig. 3:**
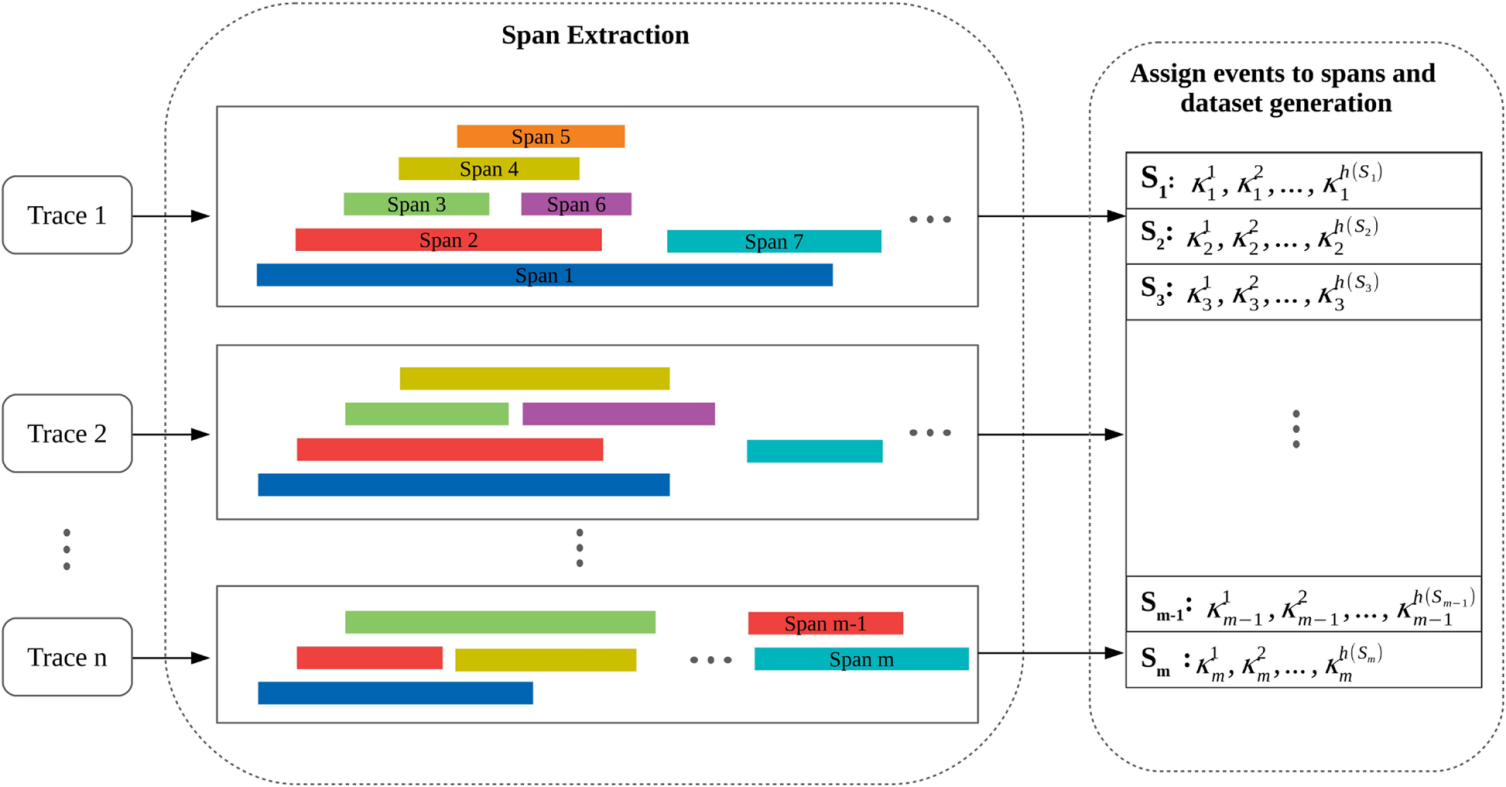
Illustration of the process for creating the training dataset from multiple traces

In order to train a model which is able to detect performance anomalies as well as release-over-release degradations, a massive training dataset is required to cover as many normal patterns of keys as possible. Actually, the training data *Γ* correspond to entries of traces obtained from the execution of previous stable releases of an application. Figure 3 summarizes how to create such a dataset. After collecting *n* different traces, each of them is processed, so as that all the spans associated with each trace are individually extracted from *Γ*. Next, for each span, its sequence of events is collected and stored in *S*_*i*_, for *i*=1,…,*m*. In our framework, each sequence *S*_*i*_ is represented by its corresponding keys $\kappa _{i}^{1},\ldots,\kappa _{i}^{h(S_{i})}$, where $\kappa _{i}^{k}$ represents the *k*-th key in the sequence *S*_*i*_, and *h*(*S*_*i*_) indicates the length of sequence *S*_*i*_.

#### **Extracting spans**

In the following, we describe how spans are extracted from an LTTng trace. LTTng uses tracepoints designed to hook mechanisms that can be activated at runtime to record information about a program execution. Tracepoints are placed inside the code by developers or debuggers to extract useful information without the need of knowing the code in-depth. Hence, one can expect to encounter different event types in trace data, indicating the beginning or the end of a span, or any other operation.

**Fig. 4:**
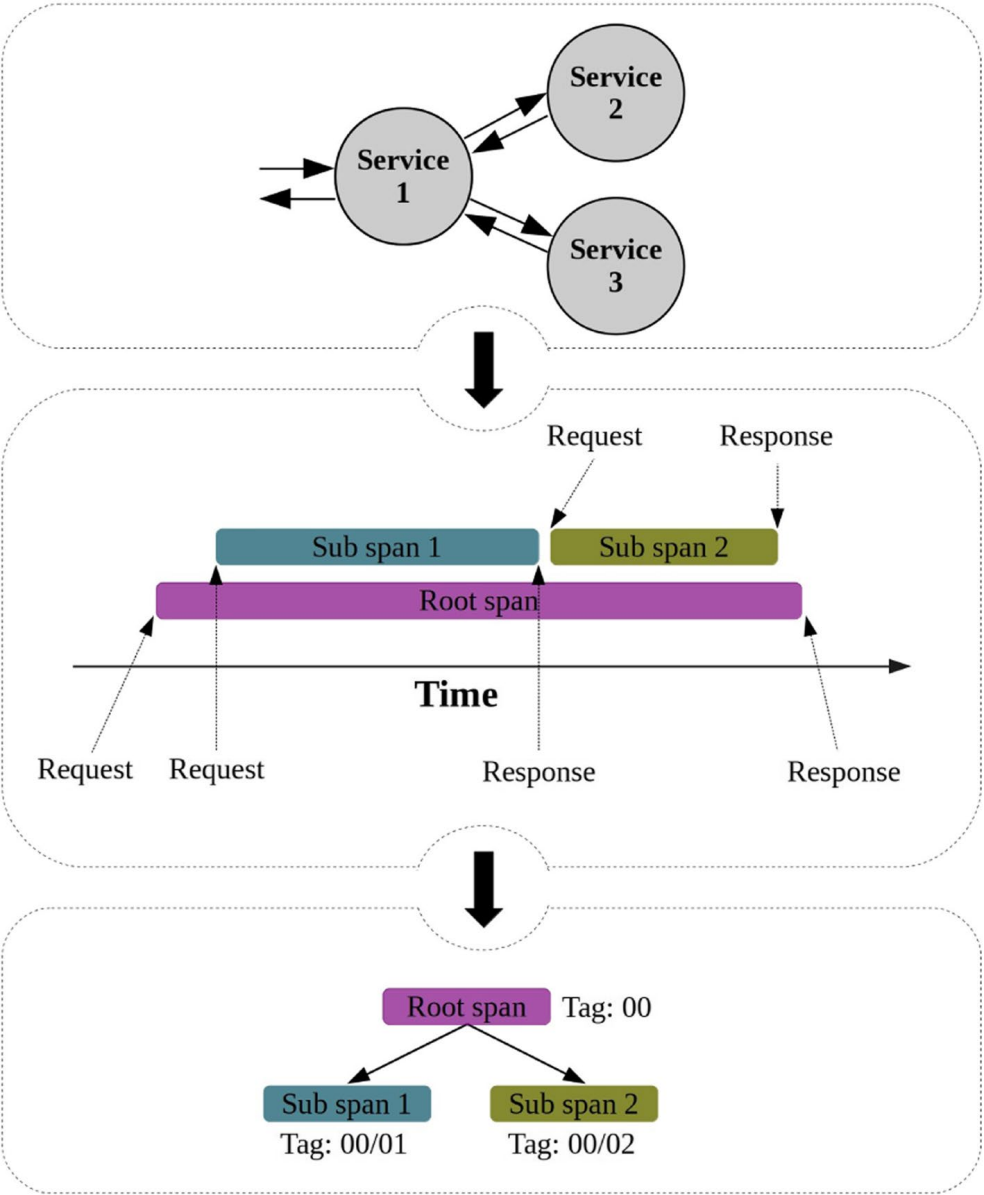
The structure of a span and its sub-spans in a distributed trace

Requests and responses are the two types of events we consider for extracting spans. Each span starts with a request and ends with a response. In addition, the request and response associated with a span possess the same tag. For example, a request with tag 00 indicates the start of a span, whereas a response with the same tag marks its end. Moreover, many sub-spans may be generated during a span’s lifetime since a service may communicate with other services to answer a demand. Similar to spans, sub-spans are created with a request and a response that share the same tag. Besides, the parent’s tag of each sub-span is embedded into the children’s tag. For example, 00/01 indicates a sub-span whose parent is represented by the 00 tag. As shown in Fig. 4, each span and its sub-spans form a tree. Yet, each span can be displayed as a sequence of requests and responses sorted by their timestamp. In the example of Fig. 4, this sequence would be *S*={*Req*,*Req*,*Resp*,*Req*,*Resp*,*Resp*}.

#### **Construction of sequences of keys**

In addition to requests and responses, many other userspace and kernel events happen during each span. After collecting all spans, all events in *Γ* are processed, and assigned to the span to which they belong. The appropriate span for each event is found by comparing the event’s arguments (e.g., TID and PID) with the arguments of the events that have been assigned to the spans. Once the appropriated span is identified, the event is placed in the sequence according to its timestamp. In the example of Fig. 4, if an event happens right after the first request, the resulting sequence becomes *S*={*Req*,*Event*,*Req*,*Resp*,*Req*,*Resp*,*Resp*}. This process is repeated for all events so that a set containing all sequences is obtained, where each sequence refers to a span.

The previous paragraph explained how sequences are extracted from a trace. Now, we explain how the arguments of events are used. Whenever a specific tracepoint is encountered at runtime, an event is produced with its arguments such as a name, timestamp, and possibly many others. Event arguments such as process name, message, and event type contain important information to increase detection quality.

The scope of this work is limited to the arguments that are common to all events. In our experimental traces, event name, process name (Procname), Thread ID (TID), Process ID (PID), timestamp, message, and event type are present in most of the events. We divided the events into two categories: 1) requests/responses, and 2) other events. Table 1 lists the arguments we selected for each category of events. The key for requests and responses is created using the name, type, tag, and procname arguments. Event type specifies whether the event is a request or a response, and tag specifies the span or sub-span to which the event belongs. The second category of general events uses the event name, procname, and message arguments to compose the keys. Thus, the resulting keys are all textual strings, and *V*={*v*_1_,*v*_2_,...,*v*_*d*_} denotes the set of all possible unique keys.

**Table 1:** The categories of events and the arguments used by our framework

Event category	Argument	Type
**Request/Response**	Event name	string
	Event type	string
	Tag	string
	Procname	string
**Others**	Event name	string
	Procname	string
	Message	string

Although simple, extending our framework to a new argument may require a much larger dataset depending on the number of values that argument may have. To illustrate, let us suppose we use only one argument to create keys, and that this argument has *β*_1_ different values. In this case, only *β*_1_ unique keys are created (*d*=*β*_1_). If another argument with *β*_2_ different values is then used, *β*_1_×*β*_2_ unique keys are obtained (*d*=*β*_1_×*β*_2_). Thus, each time a new argument with *β*_*i*_ different values is considered, the number of unique keys increases *β*_*i*_ times.

### Analysis module

In microservice environments such as our experimental application, events are expected to occur in a particular order. Actually, the keys in the sequences obtained by the data extraction module must follow specific patterns and grammar rules similar to the ones found in natural languages. Hence, only a few possible keys can appear as the next key in a sequence following a specific set of keys. The training dataset in our experiments includes normal sequences of keys obtained from previous stable releases of the application. In this section, we review the machine learning model we have proposed to distinguish normal patterns from abnormal ones. We adopted an LSTM network to model this sequence to word problem given its success for modeling text prediction and other similar natural language processing tasks. This model learns the probable keys at the moment *t* according to the previously observed sequences of keys. Later in the detection phase, the model determines which events in a sequence do not conform to normal patterns.

**Fig. 5:**
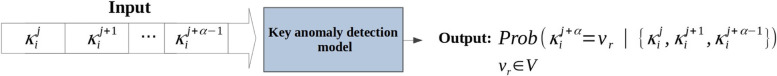
The overview of our anomaly detection model

We modeled the anomaly detection problem on our sequences of keys as a multi-class classification problem for which the input length *α* is fixed. Remark that the sequences obtained by the data extraction module are of different lengths. Multiple sub-sequences of fixed size are hence obtained by considering a window of size *α* over the larger sequences. It should be noted that sequences smaller than *α* are very rare in our dataset. These small sequences are related to light operations that are often not prone to performance anomalies. Consequently, they are simply ignored by the analysis module. Let *V*={*v*_1_,*v*_2_,...,*v*_*d*_} be the set of all possible unique keys, where each key *v*_*i*_ defines a class. From a sequence of size *h*(*S*_*i*_), *h*(*S*_*i*_)−*α* subsequences are analyzed. Thus, for each sequence *S*_*i*_, the input of the model is denoted by $X_{i}^{j}={\kappa _{i}^{j},\kappa _{i}^{(j+1)},...,\kappa _{i}^{(j+\alpha -1)}}$ and the output is expressed by $Y_{i}^{j} = \kappa _{i}^{(j+\alpha)}$, where *j*∈1,...,*h*(*S*_*i*_)−*α*. Sequences represent a part of a task’s execution path in which keys happen in a particular order. Hence, for each $X_{i}^{j}$, $Y_{i}^{j}$ can only take a few of the *d* possible keys from *V* and is dependent on the sequence $X_{i}^{j}$ that appeared before $Y_{i}^{j}$. In other words, the input of the model is a sequence of *α* recent keys, and the output is a probability distribution over the *d* keys from *V*, expressing the probability that the following key in the sequence is *v*_*r*_∈*V*. Eventually, a model of the conditional probability distribution $Prob(\kappa _{i}^{j+\alpha }=v_{r} | \left \{\kappa _{i}^{j},\kappa _{i}^{(j+1)},...,\kappa _{i}^{(j+\alpha -1)} \right \}), v_{r} \in V$ is made after the training. Figure 5 shows an overview of the described anomaly detection model.

**Fig. 6:**
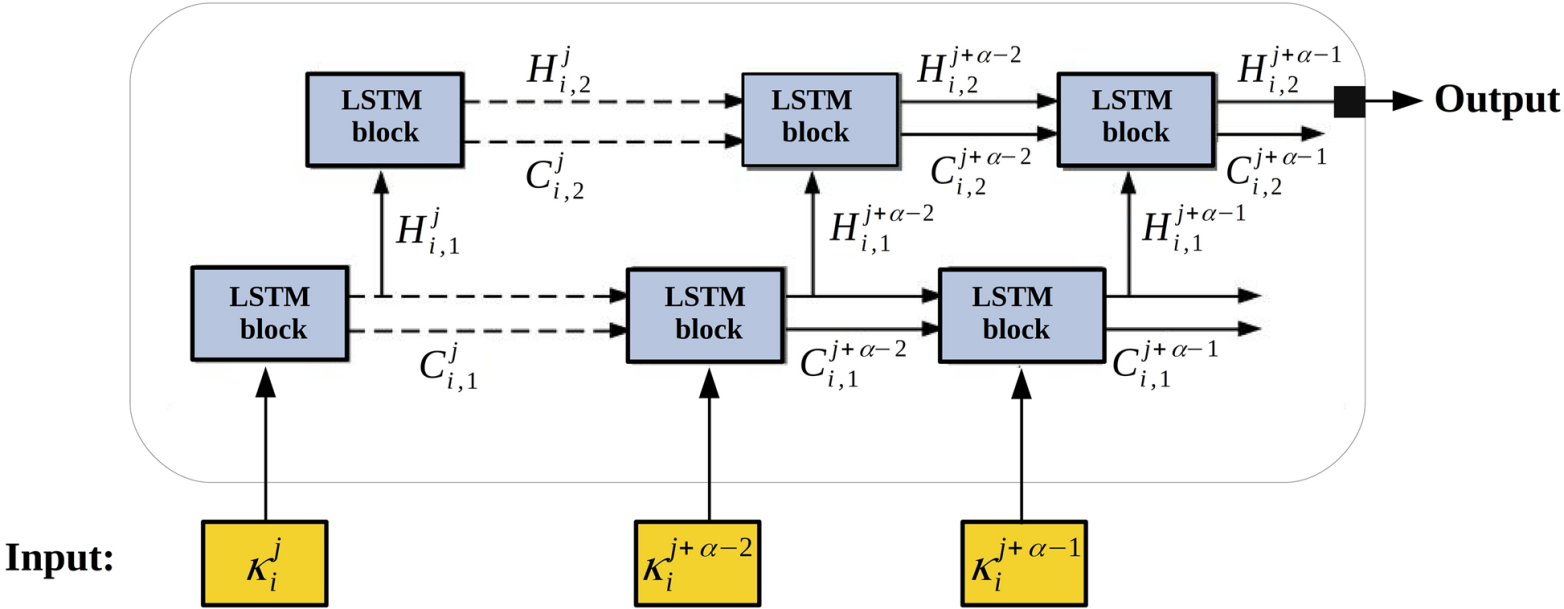
The architecture of the LSTM network we used in our anomaly detection framework

An LSTM network is employed to learn the probability distribution $Prob(\kappa _{i}^{j+\alpha }=v_{r} | \left \{\kappa _{i}^{j},\kappa _{i}^{(j+1)},...,\kappa _{i}^{(j+\alpha -1)} \right \})$ that maximizes the probability of the training sequences. The architecture of this LSTM network is shown in Fig. 6. Each layer contains *α* LSTM blocks, where each block processes a key of the input sequence. LSTM blocks have a cell state vector *C* and a hidden vector *H*. Both values are moved to the next block to initialize its state. The values of input $\kappa _{i}^{q}$ and $H_{i}^{q-1}$, for *q*∈{*j*,*j*+1,...,*j*+*α*−1}, determine how the current input and the previous output affect that state. They indicate how much of $C_{i}^{q-1}$ (the previous cell state) holds in the state $C_{i}^{q}$. They also influence the construction of the output $H_{i}^{q}$. Our deep LSTM neural network architecture includes two hidden layers in which the hidden state of the previous layer is used as the input of each corresponding LSTM block in the next layer.

During training, appropriate weights are assigned to input so that the final output of the LSTM provides the desired key. The categorical cross-entropy loss function [41] is used as the loss function for the designed multi-classification task. Then, a standard multinomial logistic function is applied to translate the last hidden state into the probability distribution $Prob(\kappa _{i}^{j+\alpha }=v_{r} | \left \{\kappa _{i}^{j},\kappa _{i}^{(j+1)},...,\kappa _{i}^{(j+\alpha -1)} \right \}, v_{r} \in V)$.

In the detection phase, the trained model is used to analyze unseen tracing data. This trace can be obtained from an old or a new release of the software. Like what was done to collect the training data, spans are extracted and then converted into sequences of different lengths. Therefore, from a sequence of size *h*(*S*_*i*_), *h*(*S*_*i*_)−*α* subsequences are obtained, and *h*(*S*_*i*_)−*α* probability distributions are predicted. The model predicts the probablity distribution $Prob(\kappa _{i}^{j+\alpha }| \left \{\kappa _{i}^{j},\kappa _{i}^{(j+1)},...,\kappa _{i}^{(j+\alpha -1)} \right \})= \left \{ v_{1} : p_{1}, v_{2}:p_{2},..., v_{d} : p_{d} \right \}$, where *p*_*j*_ describes the probability of *v*_*j*_ to appear as the next key value. Then, $\kappa _{i}^{j+\alpha }$ is marked as an unexpected key if the probability of the real seen value of $\kappa _{i}^{j+\alpha }$ is less than the confidence threshold of 0.5.

## Evaluation

In the following, we evaluate the proposed technique by analyzing a microservice-based application. First, the experimental setup and dataset generation are explained in “Experimental setup and dataset generation” subsection. Then, in “Evaluation of the anomaly detection framework” subsection, we evaluate the performance of our model. “Analysis of practical use-cases” subsection analyzes some practical use-cases and examines the success of our framework in locating the anomalies we injected into the system through various simulated scenarios. Finally, in “Root cause analysis” subsection, we explain how the scripting feature of Trace Compass as well as different views can assist experts to find the root cause of anomalies.

### Experimental setup and dataset generation

We deployed the target microservice environment (developed by Ciena Co.) on a virtualized platform with two nodes, each equipped with two cores Intel Core Processor (Broadwell, IBRS), and 4 Gb of RAM. An Oracle Linux server was installed on both nodes. Moreover, LTTng was employed on each of them to send the tracing data to the manager. The manager VM benefits from the LTTng-relayd daemon, which is responsible for receiving trace data from remote LTTng daemons.

In order to create the training data, 12 traces with duration of 5 to 10 minutes were obtained from the previous stable releases of the studied software. After removing incomplete spans, a total of 61709 spans were extracted. The dictionary of unique keys collected from the training data contains 4028 unique keys.

Our data collection module has been implemented using python and the Trace Compass Scripting feature [40]. Furthermore, we employed PyTorch to implement the LSTM network^1^. Finally, the model was trained on a server with two Intel(R) Xeon(R) Bronze 3104 1.70GHz CPUs and NVIDIA TITAN V graphic cards.

### Evaluation of the anomaly detection framework

As mentioned earlier, the analysis module models the anomaly detection problem on the sequences of keys as a multi-class classification problem. Our training dataset is composed of the sequences obtained from 12 previous stable releases of the studied software. A total of 61709 spans were extracted from these 12 traces and approximately 5 million training sequences were obtained from the spans. In addition, the dictionary of unique keys collected from the training sequences contains 4028 unique keys. Thus, there exist 4028 different classes in the training dataset, each one associated to a key.

We employed multi-class evaluation metrics to evaluate the quality of our model. Accuracy is usually the first option to evaluate multi-class classification models on a biased dataset. However, the dataset obtained in this work is not balanced. Hence, we used precision, recall, and *F*_*score* instead, which are better suited for unbalanced datasets. Precision quantifies the number of positive class predictions that actually belong to the positive class. Recall measures the number of positive class predictions that have been made out of all positive examples in the dataset. Finally, the *F*_*score* can be interpreted as a harmonic mean of the precision and recall metrics. However, the computation of these metrics for a multi-class problem is different from a binary one. In multi-class classification, these metrics are obtained for each class separately. The overall *precision*, *recall* and *F*_*score* for all classes are then computed by averaging *precision*_*i*_, *recall*_*i*_, and *F*_*score*_*i*_ for the set of classes *C*_*i*_, where *i*=1,…,4028. For an individual class *C*_*i*_, the values of *precision*_*i*_, *recall*_*i*_, and *F*_*score*_*i*_ are computed as follows [21]:

1 $$\begin{array}{@{}rcl@{}} precision_{i} = \frac{TP_{i}}{TP_{i} + FP_{i}} \end{array}$$

2 $$\begin{array}{@{}rcl@{}} recall_{i} = \frac{TP_{i}}{TP_{i} + FN_{i}} \end{array}$$

3 $$\begin{array}{@{}rcl@{}} F\_score_{i} = \frac{2\times precision_{i} \times recall_{i}}{precision_{i} + recall_{i}} \end{array}$$

**Fig. 7:**
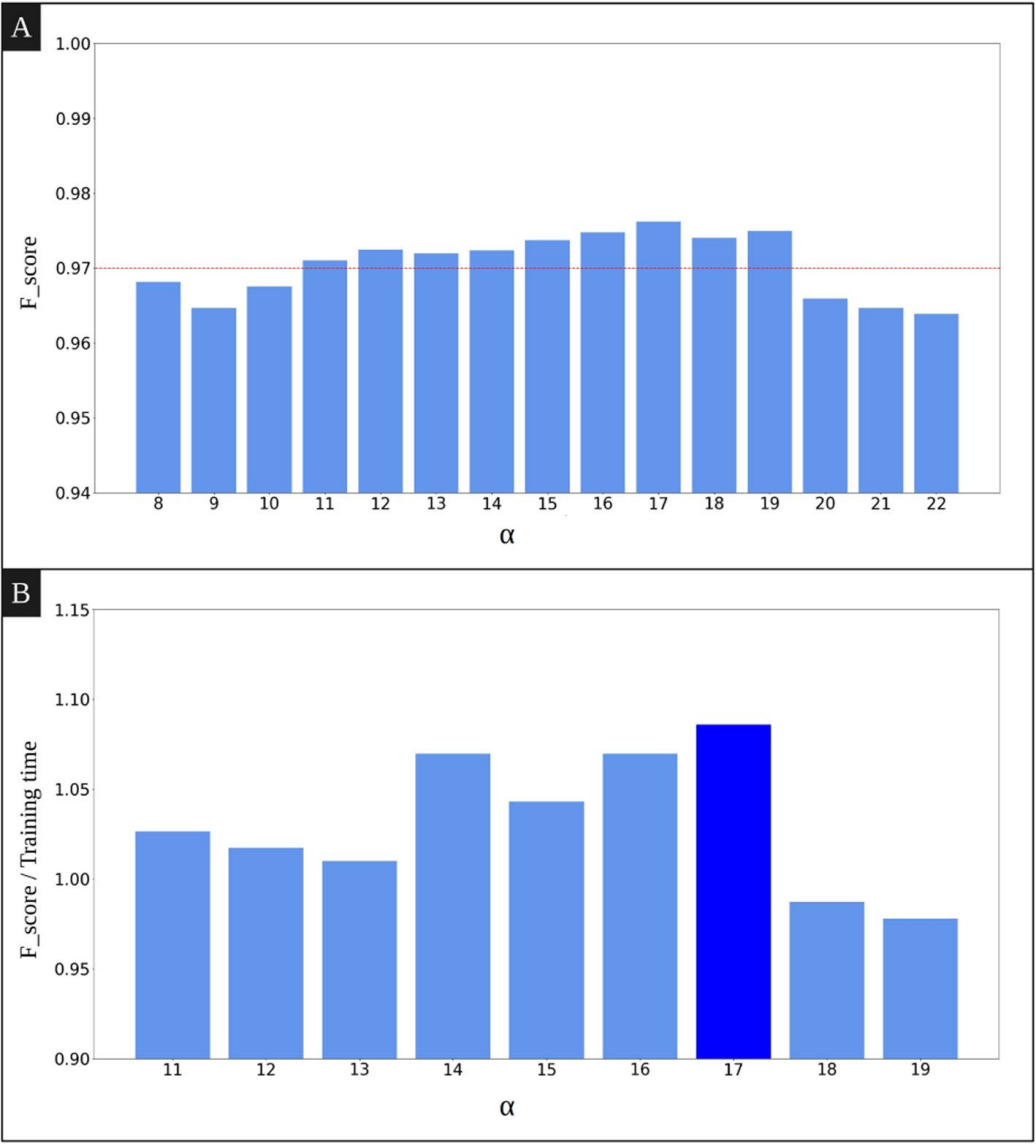
**A** *F*_*score* of the model by varying *α*. The dotted line indicates that the F_score of the model for *α* values between 11 and 19 is greater than 0.97. **B** *F*_*score*/*Training* *time* for different values of *α*. Only the values for which the F_score is greater than 0.97 are shown in this figure

In order to create the final model, the hyperparameters must be tuned. As previously explained, the sequences obtained from the spans have different lengths, but the input length of the model is fixed as *α*. Therefore *h*(*S*_*i*_)−*α* sub-sequences can be obtained by taking a window of size *α* over a sequence of size *h*(*S*_*i*_). The value of this hyperparameter affects both the training time and the performance of the model. So, we tried to choose a value for *α* that would lead to a highly effective model in a reasonable training time. For this purpose, we measured the f_score and training time of the obtained models by selecting *α*∈{8,9,..,30}. The smallest possible *α* value is 8 since that corresponds to the minimum sequence length in the training data. Figure 7(A) shows that for *α* values greater than 19, the *F*_*score* of the model begins to decrease. We omitted the results for 23≤*α*≤30 to make the figure clearer. Figure 7(B) presents the *F*_*score*/*training* *time* for the best found *α* values. According to the results, *α*=17 achieves the best classification results in terms of training time and *F*_*score*. This value is therefore selected for the remaining of our experiments.

After selecting the hyperparameter *α*, we evaluated the quality of our model through 10-fold cross-validation. In 10-fold cross-validation, the dataset is divided into ten subsets of approximately equal size. One of the subsets is reserved for testing, while the remaining subsets are used for training. This process is repeated 10 times, and the results are averaged over each one of 10 different tested subsets. Results of evaluating our model with 10-fold cross-validation are listed in Table 2. Our proposed method demonstrates excellent performance with a *F*_*score* of 0.9759. It should be noted that our dataset contains approximately 5 million sequences and 4 thousand unique keys.

**Table 2:** Results of evaluating our model with 10-fold cross- validation

**Precision**	0.9774	
**Recall**	0.9760	
**F_score**	0.9759	

### Analysis of practical use-cases

In this subsection, a newer release of the application was investigated to evaluate the model on detecting possible performance degradations and anomalies. For this purpose, we examined three different scenarios. In the first scenario, the regular execution of the application, i.e., without any anomaly injection, was analyzed to determine where and why the new release did not follow the normal patterns learned by the model. In the other two scenarios, we investigated the performance vulnerability of the new release when an external factor disrupts fair access to system resources. To simulate such attacks, a significant CPU load on the multi-core nodes was generated as the second scenario by continuously compressing and decompressing a stream of random data (zip bombs). Finally, in the third scenario, disk stress was injected into the nodes by creating a file and then using a loop to copy it repeatedly.

After collecting the tracing data for each of the mentioned scenarios, the data collection module extracted the spans from these tracing files and created the sequences of keys. For all input subsequences, the model determines whether the key that appeared in the sequence right after the input subsequence is probable to happen or not. The model marks that key as an *unexpected key* if it predicts that the probability of that key in the sequence is lower than the confidence threshold of 0.5.

Table 3 reports the number of detected unexpected keys along with the total number of predictions made by our model for three scenarios. As expected, the number of detected unexpected keys in the first scenario is less than in the other two other scenarios, where CPU and disk stress were injected into the system. The injected load in the second and third scenarios has made the application behave much differently. The first scenario reveals how the changes applied to the application by developers in a newer release may affect the execution’s path of the application.

**Table 3:** The number of detected unexpected keys along with the total number of predictions made by our model for the three scenarios

	Number of detected unexpected keys	Number of predictions
**Test data (Scenario 1)**	32043	518503
**Test data (Scenario 2)**	65507	489593
**Test data (Scenario 3)**	66978	453781

Our proposed framework, however, does not signal keys as anomalous as soon as an unexpected key is detected. It also takes into consideration the frequency of unexpected keys over the monitored period of time. Once a high frequency of unexpected keys is identified, that sequence of keys is highlighted for further investigation by developers or system experts. This is intended to reduce troubleshooting time, as the developers can examine few specific intervals instead of looking at large amounts of tracing data, which might include thousands of system events. As we show next, the output of the model can be examined from two different perspectives.

#### **System-based anomaly detection**

**Fig. 8:**
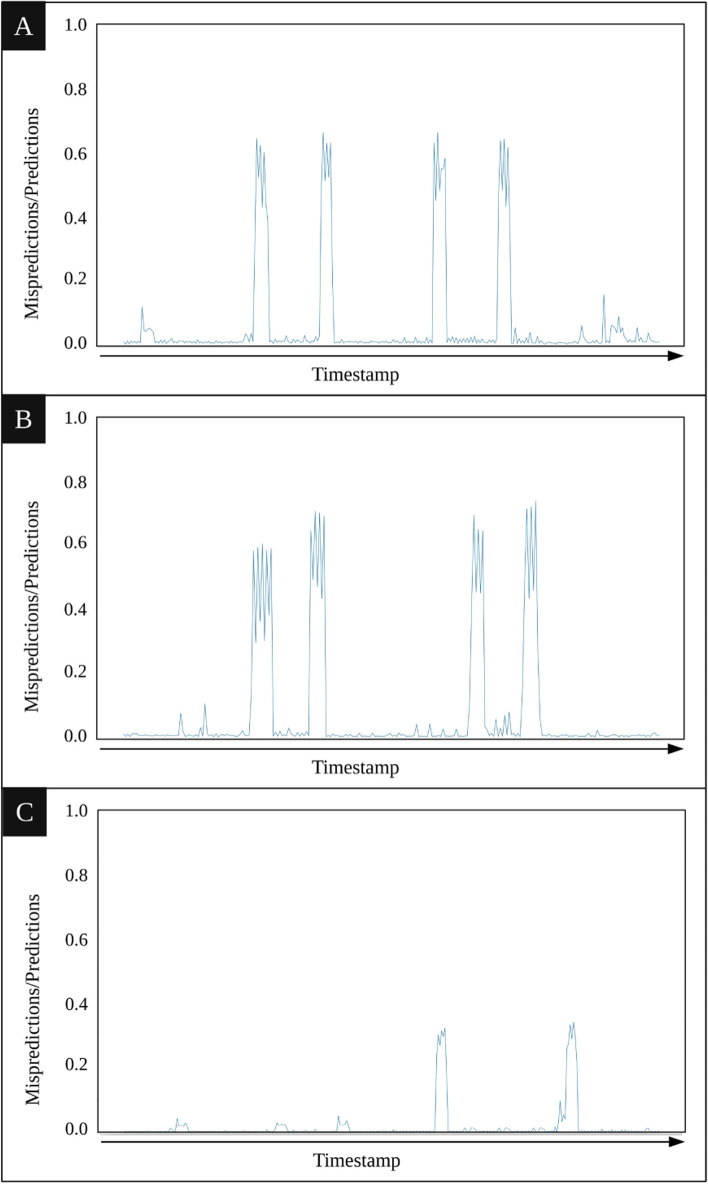
This Figure depicts the Likelihood of detecting unexpected keys over the traces obtained from the three mentioned scenarios. **A** In this scenario, CPU-related anomalies were injected into the system. **B** In this scenario, disk-related anomalies were injected into the system. **C** In this scenario, a new release of the application without injecting anomaly was investigated

In system-based anomaly detection, the entire execution is examined regardless of the span to which each unexpected key belongs. To illustrate, let us consider traces of 5 minutes divided into small time intervals of 1 second. The chart displayed in Fig. 8 shows the rate of detecting unexpected keys, i.e., number of detected unexpected keys divided by the total amount of predictions, computed in each of the monitored intervals for the three tested scenarios. They reveal the intervals in which more unexpected keys have detected, and are hence, more likely to represent anomalies. This view helps developers to focus only on the areas prone to anomalies. The peaks in Figs. 8(a) and 8(b) correspond exactly to the moments the anomalies were injected into the system, being correctly discovered by our framework. Figure 8(c) includes a smaller number of peaks with lower heights. The observed peaks indicate the moments in which the new release did not follow the normal behavior of the previous ones.

#### **Service-based anomaly detection**

In service-based anomaly detection, we detect anomalous spans. Unlike system-based anomaly detection, in which we examine the entire execution, service-based anomaly detection identifies spans with a high rate of unexpected keys. The rate of unexpected keys for each span correspond to the rate of unexpected keys in the sequence associated with that span.

**Fig. 9:**
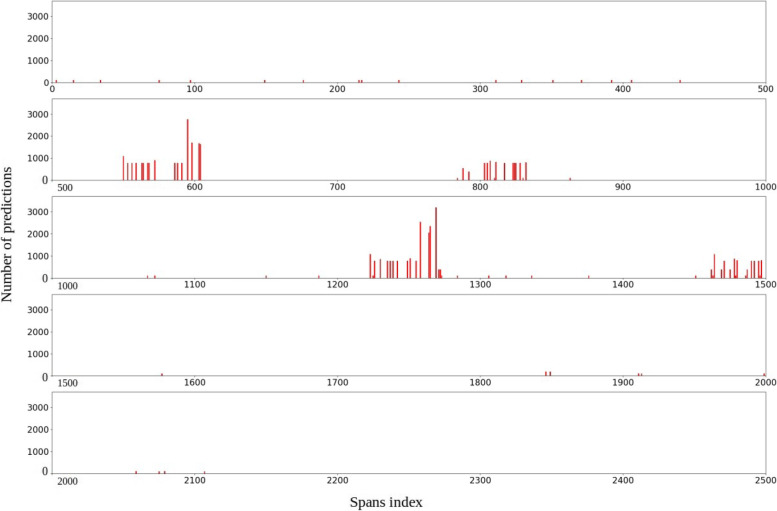
The anomalous spans that appeared during the trace of the second scenario where each span has been drawn with a bar

A span for which the rate of unexpected keys is greater than 0.5 is marked as an anomalous span. The chart of Fig. 9 depicts the anomalous spans detected by our framework during the test trace obtained from the second scenario. Spans are numbered according to their start time and are shown with a red bar. In Fig. 9, the x-axis shows the spans index and the y-axis indicates the rate of unexpected keys. From this figure, we observe that many anomalous spans have been detected when anomalies were injected in the system. This view enables developers to filter a trace based on the anomalous spans tag, that merits further investigation.

### Root cause analysis

Using Trace Compass in our framework provides the developers an in-depth perception of what happens during a trace, especially in the presence of anomalies. Trace Compass is already used by many companies in the field of performance analysis. We have converted the output of our anomaly detection model to Google’s Trace Event format to be able to investigate the root causes of the identified anomalies. Our output in this format contains a set of events, each of which equivalent to an event in the original trace. However, three new fields have been added to each event. Field category determines whether the event is identified as unexpected. In addition, each event keeps the tag of the span in which it is located, and finally, another field shows if the related span is abnormal or not.

**Fig. 10:**
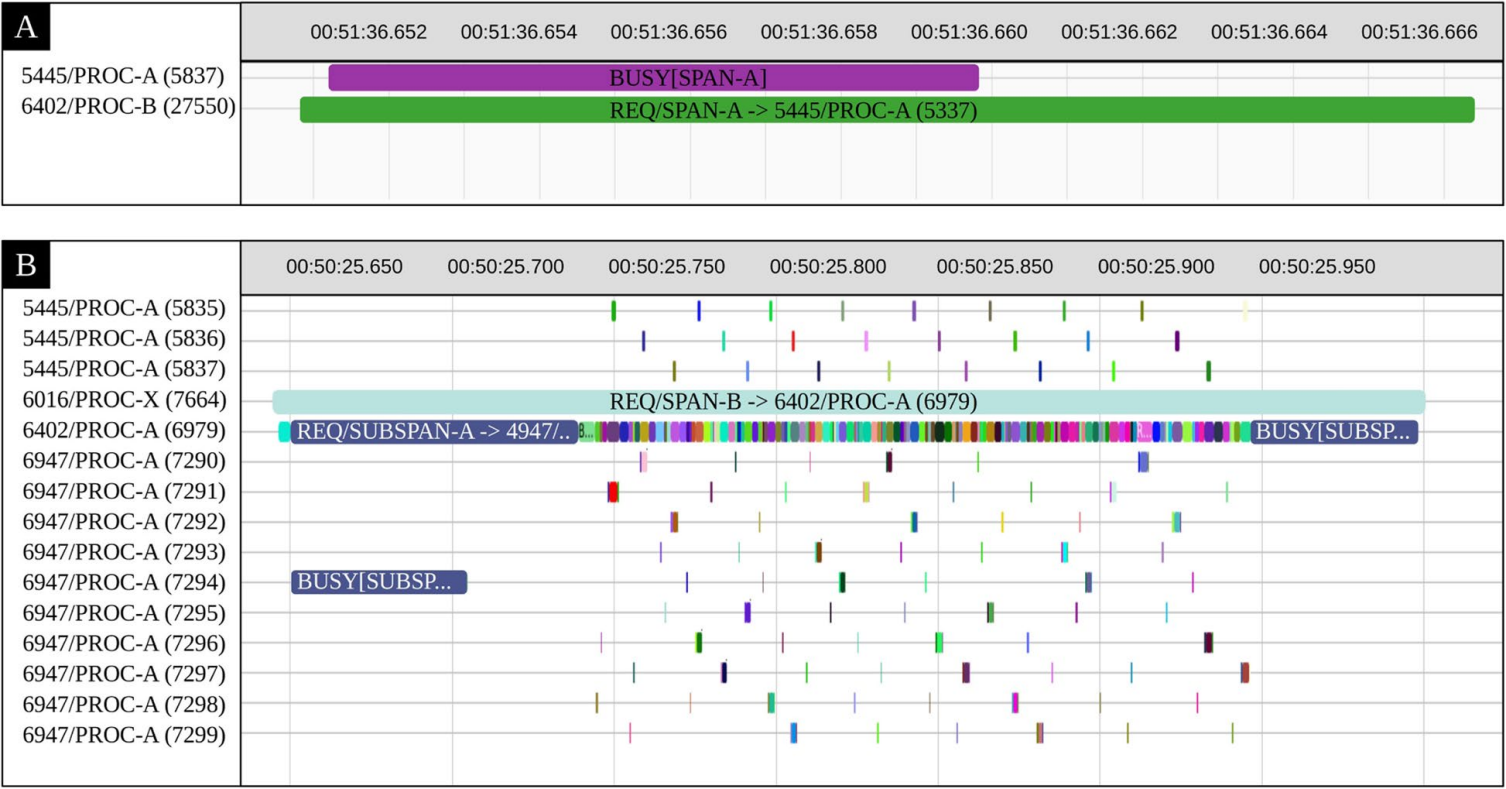
This figure presents the time chart generated by our script in Trace Compass, which helped to find the cause of anomalies in our test traces (due to Ciena’s security rules, we have changed the original names of the processes in these screenshots). **A** A sample of a normal span. **B** A sample of an anomalous span where the PROC-X is the caused of the problem

To understand the cause of the anomalies in the introduced test traces, we provided a script that separates all the processes in the trace thereby displaying them with different colors in a time chart like illustrated in Fig. 10. This time chart can be zoomed in and out in particular areas. Furthermore, the time axis in this time chart is aligned with other views and tables that support automatic time axis alignment, such as the editor view that presents the events in a tabular format, or the statistics view that displays the various event counters. More detailed data can be computed from the trace as the user zooms in the time chart or filters events in the editor table.

Figure 10(a) shows the structure of a sample normal span. In Fig. 10(b), the trace was filtered based on the tag of one of the anomalous spans. Interestingly, the provided Trace Compass script could successfully find the cause of a latency issue in the target application that has been detected by our anomaly detection model, then pointing out to the process that caused this problem. This process was present in many other abnormal spans as well. These results demonstrate the effectiveness of our proposed framework in locating anomalies and finding their root causes.

## Conclusion

In recent years, advances in technology and computing power have led to the emergence of complex and large-scale software architectures like microservices and IoT devices that speed up different tasks. Despite all the advantages of these systems, several factors, such as their distribution in the network, the use of different technologies, their short life, software bugs, hardware failures, and resource contentions, make them prone to the rise of anomalous system behaviors. Besides, available performance monitoring and analysis tools have many shortcomings. In this research, a general-purpose NLP-based anomaly detection framework was presented for detecting abnormal behaviors and release-over-release regressions in microservice environments.

The proposed framework is based on recording streams of events in the tracing module, sending them to the data extraction module so as to create sequences of keys, which are finally analysed using a deep LSTM model. This framework is general enough to work with any application since it benefits from an all-purpose open-source distributed tracing tool to collect event sequences. Notably, our framework learns a representation of the event names along with other arguments to remedy the limitations of other methods. In addition, the whole system needs no prior knowledge which facilitates the collection of training data.

Extensive experiments on real datasets confirm the effectiveness of our framework in detecting abnormal behaviours in microservice environments. Our framework is also projected to help in the root cause analysis of system issues, which can be performed through various plots and scripts that we have provided in Trace Compass. In addition, the proposed framework is easy to deploy and use in practice since no particular assumptions and settings are considered in the data collection. Taken together, these findings suggest that our framework is an effective tool to reduce troubleshooting time by directing the developer to the most relevant problem sites of interest.

In the future, we will examine the impact of employing kernel tracing on the proposed approach. We will also extend this work to use other events arguments. Finally, it would be interesting to investigate other NLP techniques to improve detection performance.

## Data Availability

The data and materials are available from the corresponding author on reasonable request.

## Footnotes

1 The implementations of the data collection module and the detection module are available in https://github.com/kohyar/LTTng_LSTM_Anomaly_Detection.
